# Role of the DNA Damage Response in Human Papillomavirus RNA Splicing and Polyadenylation

**DOI:** 10.3390/ijms19061735

**Published:** 2018-06-12

**Authors:** Kersti Nilsson, Chengjun Wu, Stefan Schwartz

**Affiliations:** Department of Laboratory Medicine, Lund University, 221 84 Lund, Sweden; Kersti.Nilsson@med.lu.se (K.N.); troy_chengjun.wu@med.lu.se (C.W.)

**Keywords:** papillomavirus, splicing, polyadenylation, SR proteins, hnRNP C, BRCA1, BCLAF1, TRAP150, DDR, U2AF65

## Abstract

Human papillomaviruses (HPVs) have evolved to use the DNA repair machinery to replicate its DNA genome in differentiated cells. HPV activates the DNA damage response (DDR) in infected cells. Cellular DDR factors are recruited to the HPV DNA genome and position the cellular DNA polymerase on the HPV DNA and progeny genomes are synthesized. Following HPV DNA replication, HPV late gene expression is activated. Recent research has shown that the DDR factors also interact with RNA binding proteins and affects RNA processing. DDR factors activated by DNA damage and that associate with HPV DNA can recruit splicing factors and RNA binding proteins to the HPV DNA and induce HPV late gene expression. This induction is the result of altered alternative polyadenylation and splicing of HPV messenger RNA (mRNA). HPV uses the DDR machinery to replicate its DNA genome and to activate HPV late gene expression at the level of RNA processing.

## 1. Introduction

Human papillomaviruses (HPVs) are small DNA viruses that infect the keratinocytes of squamous and mucosal epithelia [1,2]. Thought to precede the amniotes (reptiles, birds and mammals), they are highly adapted to their host and most HPV infections are asymptomatic and resolve spontaneously. However, in rare cases, some HPV infections persist and cause disease such as warts and cancer. Approximately 50% of all virus-associated human cancers are caused by HPV [3]. This is largely attributed to a subset of sexually transmitted HPVs that cause anogenital and head and neck cancer. HPV16 is the most prevalent of the cancer-associated HPV types [4,5]. Knowledge of the HPV gene expression program is important to understand how HPV interacts with the infected cell in a manner that causes long-term persistence and cancer.

### The Life Cycle of HPV

**Figure 1:**
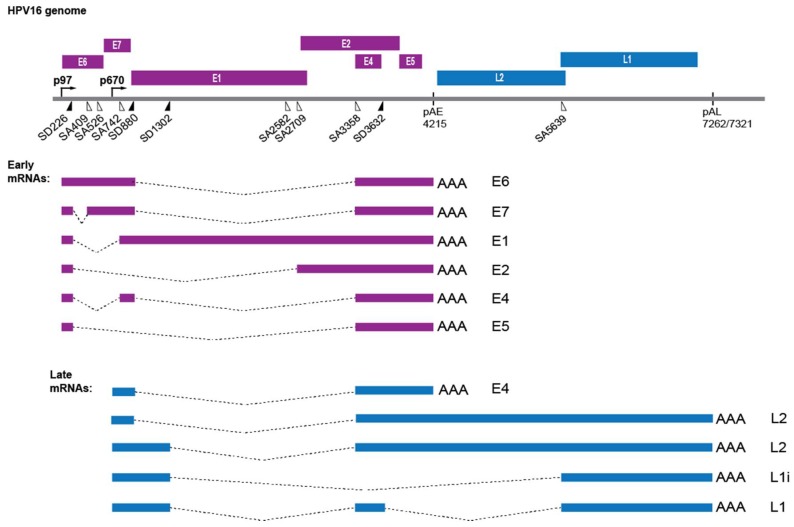
Schematic representation of the HPV16 genome. HPV16 early genes E1, E2, E4, E5, E6 and E7 and HPV16 late *L1* and *L2* genes are indicated. HPV16 early promoter (p97), late promoter (p670) and early (pAE) and late (pAL) polyadenylation signals are shown. Filled triangles represent 5′-splice sites and open triangles represent 3′-splice sites. Splice sites SD226, SA409, SA526 and SA742 are used exclusively by early mRNAs; SD3632 and SA5639 are used exclusively by late mRNAs; and splices sites SD880, SD1302, SA2582, SA2709 and SA3358 are used both by early and late mRNAs. A subset of HPV16 alternatively spliced early mRNAs and late mRNAs are shown.

The HPV genome is about 8 kb in size and exists as an episome, a circular genome with independent replication [6,7]. The viral genome is associated with histones in a manner that is highly similar to human chromatin organization [8]. The HPV16 coding region contains at least six early (E) genes (Figure 1), which are expressed in the lower and mid layers of the infected epithelium. The HPV genome also encodes two late (L) genes, which encode the L1 and L2 structural proteins that are expressed only in terminally differentiated keratinocytes in the upper part of the epithelium (Figure 1) [9].

The life cycle of HPV is coupled to the differentiation program of the keratinocyte, which results in an ordered expression of the viral genes [10]. HPV has no means of replicating its own DNA genome and is totally dependent on the DNA replication machinery of the host cell. Therefore, infection starts by HPV gaining access to the actively dividing cells in basal layer of the epithelium. Replication of the viral genome is divided into three phases; establishment-, maintenance- and productive-replication [7]. In the basal layer, the genome is amplified to a low copy number during establishment replication that is followed by maintenance amplification and HPV early gene expression. E6 and E7 promote cell cycle entry and prevent p53-mediated apoptosis to delay epithelial differentiation and maintain expression of cellular replication factors [11,12,13]. HPV E1 and E2 are directly involved in HPV genome amplification [14,15]. Downregulation of E6 and E7 expression eventually allows for terminal cell differentiation, expression of the HPV late genes L1 and L2 and production of progeny virus. The HPV gene expression program is dictated by the cellular differentiation program that controls HPV gene expression at the level of transcription [16,17] and at the level of RNA processing, including alternative splicing and polyadenylation [18,19,20]. HPVs produce a plethora of alternatively spliced and polyadenylated mRNAs that are controlled by cellular- [18,19,20,21,22] and viral factors (Figure 1) [18,23]. In this review, we discuss how DNA damage response (DDR) factors that are recruited to the HPV DNA to replicate the HPV genome can also be utilized to activate HPV late gene expression at the level of RNA splicing and polyadenylation. This review focus on the most common cancer-associated HPV types of the α-genus with emphasis on HPV type 16.

## 2. Human Papillomavirus (HPV) and the Cellular DNA Damage Response (DDR)

### 2.1. HPV Employs the Cellular DNA Damage Response for Genome Amplification

**Figure 2:**
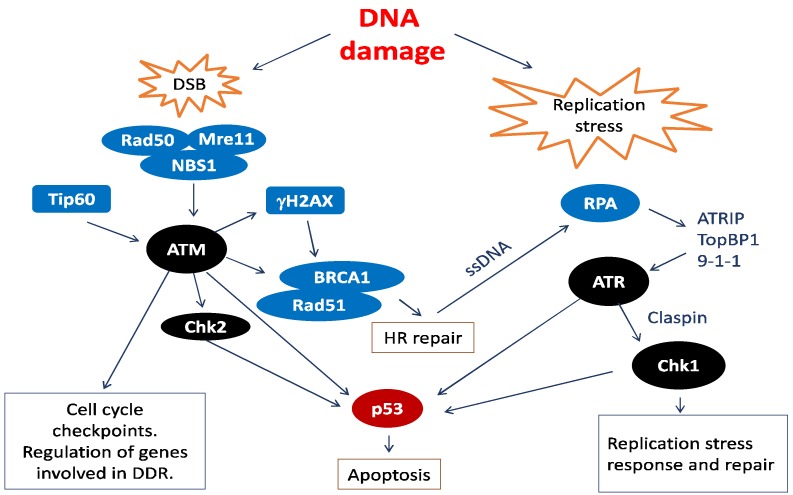
The Ataxia-Telangiectasia Mutated (ATM) and ATM and Rad3-related (ATR) signalling pathways in response to DNA damage. Double stranded breaks (DSBs) are detected by the sensory complex MRN (Mre11, Rad50, and Nbs1). The MRN complex and the acetyltransferase Tip60 activate ATM, which relays the damage signal to targets such as γH2AX, Chk2, p53, and Breast Cancer Susceptibility Gene 1 (BRCA1). γH2AX nucleates the site of damage, leading to the recruitment of several E3 Ubiquitin ligases that bring homologous repair factors (HR) such as BRCA1 and Rad51 to the site of damage. Downstream effects of the signal are cell cycle arrest, DNA repair, or apoptosis. ATR is activated in response to single stranded DNA (ssDNA) that arises when damaged DNA interfere with replication or transcription. ATR can also be activated in an ATM-dependent manner during repair of DSBs as intermediate structures during repair display ssDNA. The Replication Protein A (RPA) forms filaments on ssDNA and recruits ATRIP, the 9-1-1 complex (Rad9-Hus1-Rad1) and TopBP1 that all activate ATR. The damage signal is then passed on via Claspin and Chk1 and the DNA damage is repaired, if possible.

The integrity of the eukaryotic genome is maintained through a network collectively referred to as the DNA damage response (DDR) that senses and signals DNA damage arrests the cell cycle and activates repair mechanisms or eliminates the damaged cells through apoptosis (Figure 2). Different types of insult to the DNA are detected through unique sensors. DNA damage signals are then relayed to effector molecules in a manner similar to signal transduction pathways, including post-translational modifications such as phosphorylation [24]. The major upstream kinases in the signal transduction pathway that orchestrate the response to DNA damage are members of the phosphatidylinositol 3-kinase-related kinase (PIKKs) family and include Ataxia telangiectasia mutated kinase (ATM) and Ataxia telangiectasia and Rad3-related protein FRAP-related protein 1 (ATR) (Figure 2) [25]. ATM and ATR appear to regulate the broadest spectrum of downstream factors that contribute to the DDR (Figure 2) [26,27,28]. In addition, they induce further phosphorylation events through the activation of the Chk1 and Chk2 kinases (Figure 2) [29,30]. ATM is activated in response to double stranded breaks (DSBs) [31,32], whereas ATR is activated by the presence of single stranded DNA [25,33,34]. The downstream events in the DDR signal transduction chain include cell cycle check-points, apoptosis or DNA synthesis to restore the integrity of the DNA molecule. The latter feature of the DDR is exploited by some DNA viruses such as HPV that lacks a DNA polymerase and has evolved to employ the DDR for amplification of the viral genome.

### 2.2. HPV Proteins Perturb Cell Differentiation to Allow for Replication of HPV DNA

Keratinocytes exit the cell cycle and differentiate as they leave the basal layer. To maintain an environment that supports viral replication, HPV E7 binds to the Rb family proteins to alleviate their suppression of the cellular transcription factor E2F [12]. The liberated E2F protein activates expression of cell cycle promoting proteins. Consequently, the HPV-infected cell enters a G2-like phase in which differentiation factors and replication factors required for productive viral replication can coexist [35,36]. Meanwhile, HPV E6 targets p53 for degradation to suppress p53-mediated apoptosis that would otherwise have been elicited by the unscheduled re-entry into the cell cycle [13]. The HPV proteins E1 and E2 support initial establishment and maintenance replication of the HPV genome. HPV E1 is a DNA helicase that separates the DNA strands at the HPV origin of replication, while E2 functions by positioning E1 and the cellular replication machinery onto the HPV DNA genome [14,15]. Efficient amplification of HPV genomes requires activation of the late, differentiation-dependent HPV promoter to provide high expression levels of the HPV E1, E2 and E4 proteins. Initially, the early promoter remains active upon differentiation that allows expression also of E6 and E7. However, the HPV early promoter is subsequently shut down by the accumulated levels of the E2 protein to allow for cell differentiation and differentiation-dependent expression of the HPV late *L1* and *L2* genes.

### 2.3. DDR Factors Contribute to HPV DNA Replication

**Figure 3:**
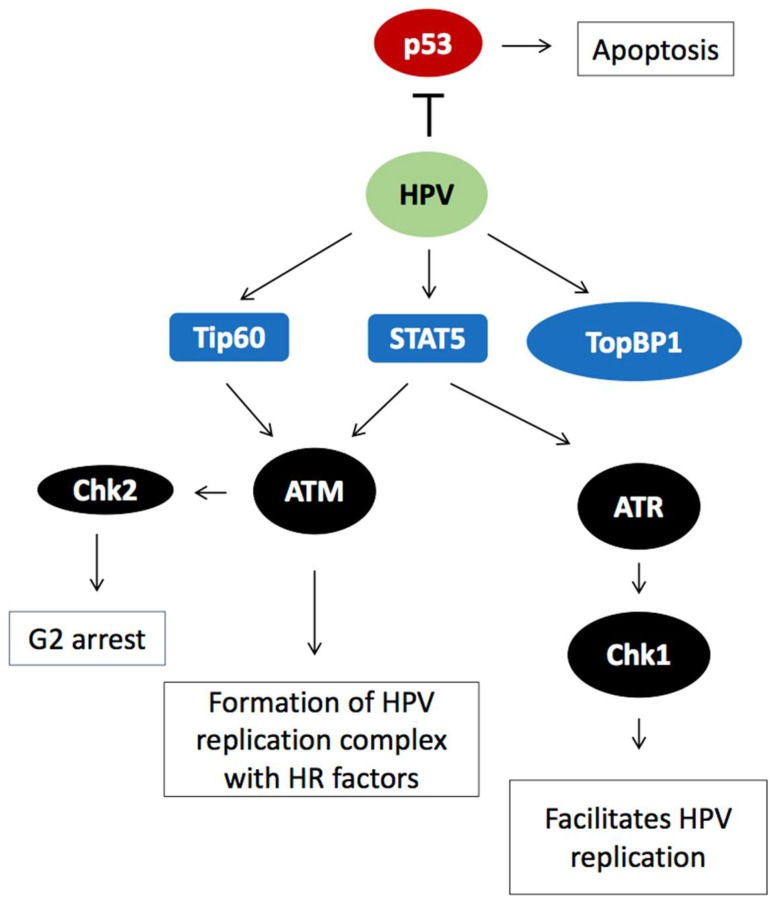
Interactions between HPV and the Ataxia-Telangiectasia Mutated (ATM) and ATM and Rad3-related (ATR) signalling during productive viral replication. HPV activates the ATM branch of the DDR to gain access to factors associated with homologous recombination. This activation occurs at least partially through the Tip60 acetyltransferase and signal transducer 5 (STAT5) that are both required for activation of ATM. In addition, the ATR branch of the DDR is activated by HPV. HPV replication foci contain TOPBP1, a protein necessary for ATR activation. The exact mechanism of ATR activation is unclear Downstream of ATM/ATR signal transduction are the kinases Chk1 and Chk2, both of which have been found in HPV replication foci and are known to be crucial for cell cycle arrest and regulation of genes needed for HPV genome amplification. To counteract the potential induction of apoptosis by the cellular DDR, HPV E6 targets p53 for degradation to inhibit apoptosis.

In addition to HPV proteins, HPV genome amplification also requires cellular proteins of the ATM and ATR branches of the DDR [37,38,39]. ATR is active during all stages of the HPV life cycle [38,39], suggesting that this branch of the DDR is necessary for initial-, maintenance- and productive-replication [3,40,41]. Further, TopBP1 that acts upstream of ATR signalling is a required component of the viral replication loci [39]. The HPV E1 and E7 proteins can independently activate ATR and Chk1 [3,38,42]. Alternatively, this activation is a consequence of the replication stress that arises from replication of the HPV genome, the unspecific DNA helicase activity of E1, the aberrant cell cycle entry created by the viral proteins or the ssDNA generated during homologous recombination (HR)-mediated productive HPV replication [43]. However, different HPV types seem to have specific effects on the ATR signalling [44]. As the signalling from the ATM and ATR branch overlap, perhaps this reflects a variable ability of HPV proteins to interact with cellular components to elicit the DDR required for genome amplification [44]. The HPV infection activates the DDR with the purpose of exploiting the DDR DNA synthesis machinery for HPV genome replication (Figure 3). However, induction of the DDR is accompanied with a risk of inducing p53-mediated apoptosis. To prevent apoptosis, the HPV E6 protein binds and degrades cellular p53 (Figure 3). ATM is also active in HPV infected cells and contributes to the productive phase of HPV DNA replication [3,39]. As the levels of HPV E1 and E2 rise in the mid layers of the HPV-infected epithelium, E1 and E2 nucleate the viral origin of replication together with cellular HR factors Rad51, BRCA1 and the MRN (MRE11, Rad50and NBS1) complex (Figure 3). These factors are all required for productive HPV DNA replication. HR mediated repair creates a large area of ssDNA that invades a sister chromatid to use a homologues sequence as template for synthesis of new DNA. Thus, HPV may specifically activate ATM to recruit HR factors as they offer high fidelity replication in G2-arrested cells upon differentiation. Alternatively, ATM activation is a result of the rolling circle replication used for the productive amplification of the viral genome [45]. The modified histone γH2AX, a hallmark of DNA damage, is also found on HPV genomes at onset of productive replication [46]. It is aiding in the recruitment of DNA repair factors to the HPV genome. Additional proteins associated with the ATR branch of the DDR, such as CHK1 and TopBP1, are also found in the HPV replication foci [3,41,47]. HPV E7 appears to increase the abundance of these factors, partly through transcriptional activation by E2F [48], partly through protein stabilization [37,39]. Activation of the DDR by E7 is also mediated by interactions with signal transducer and trans activator 5 protein (STAT5) and the Tip60 acetyltransferase (Figure 3) [38,49,50]. In conclusion, several cellular DDR factors are required for replication of the HPV DNA genome.

### 2.4. HPV Gene Regulation

The coding region of the HPV genome consists of at least two promoters, two polyadenylation signals and eight protein-coding genes (Figure 1). The early (E) genes are expressed from the early promoter and polyadenylated at the early polyadenylation signal (pAE) (Figure 1). However, early proteins E1, E2 and E4 can also be expressed from mRNAs initiated at the HPV late promoter but are polyadenylated at pAE (Figure 1). HPV late genes *L1* and *L2* are expressed from the late promoter and polyadenylated at the late polyadenylation signal (pAL) (Figure 1). To ensure efficient expression of each viral gene in a highly regulated fashion, HPV makes extensive use of alternative mRNA splicing and polyadenylation [18,19,20,21,22,51]. Although HPV uses the cellular splicing and polyadenylation machineries, the HPV genome differs from the cellular genome in that the vast majority of the HPV genome is protein coding (Figure 1). In addition, many of the HPV open reading frames (ORFs) overlap. The molecular anatomy of the HPV genome is therefore particularly challenging since RNA elements that control HPV splice sites and polyadenylation signals are likely to be situated in regions of the HPV genome that are constrained by a protein coding region, or even two overlapping protein coding regions (Figure 1) [18,19,20]. In addition, the 3′-untranslated regions of HPV encode RNA elements that control HPV mRNA stability and/or translation efficiency [52].

Expression of the HPV late *L1* and *L2* genes requires a switch to the differentiation-dependent late HPV promoter. The late promoter is located in the 5′-end of the genome, while the *L1* and *L2* genes are located in the 3′-end of the genome (Figure 1). Consequently, mRNA splicing and polyadenylation play major roles in the control of HPV late gene expression [18,19,20]. In addition to activation of the HPV late promoter, inhibition of the early polyadenylation signal pAE is required for production of pre-mRNAs encoding L1 and L2. Activation of the two suppressed, exclusively late splice sites SD3632 and SA5639 gives rise to the L1 mRNAs and is paramount for L1 and L2 expression [53,54]. High levels of the HPV16 E2 protein inhibit HPV16 early polyadenylation and E2 therefore contributes to activation of HPV16 late gene expression [55]. In addition to E2, recruitment of cellular splicing factors and RNA binding proteins is of vital importance for HPV late gene expression [22].

### 2.5. Induction of HPV Late Gene Expression by the DNA Damage Response

**Figure 4:**
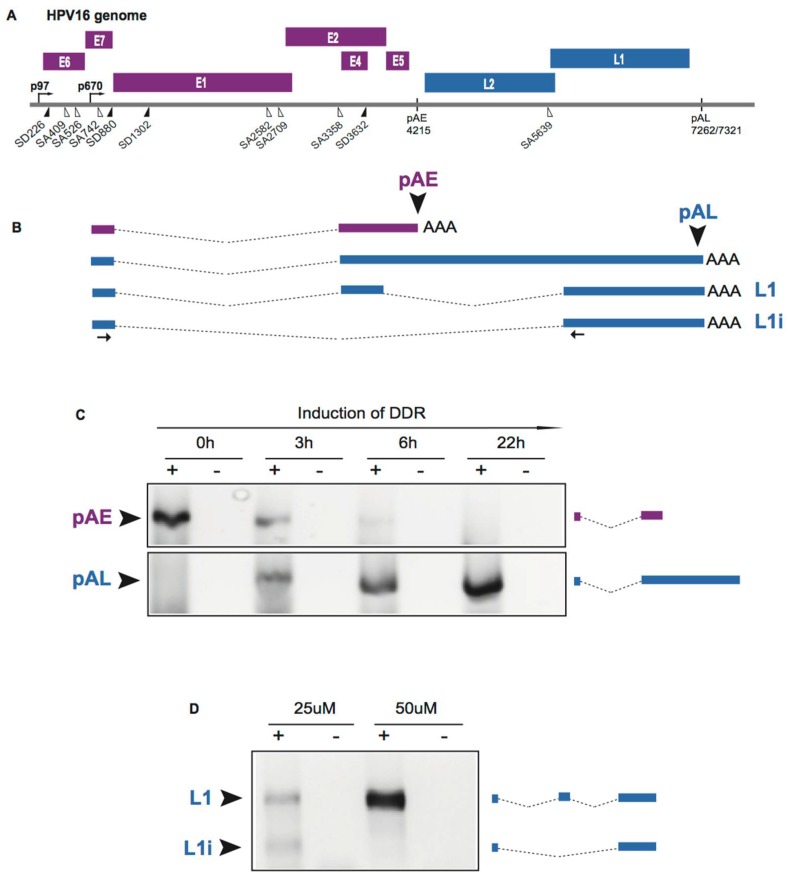
The DNA damage response alters HPV16 mRNA polyadenylation and splicing. (**A**) Schematic representation of the HPV16 genome. (**B**) Examples of alternatively polyadenylated and alternatively spliced HPV16 mRNAs. (**C**) 3′-RACE assay with primers specific for either the HPV16 early polyadenylation signal pAE, or HPV16 late polyadenylation signal pAL was performed on RNA extracted from HPV16 reporter cell line C33A2 treated with 100uM melphalan for the indicated time periods. Induction of the DNA damage response with melphalan in the HPV16 reporter cell line C33A2 inhibits HPV16 early polyadenylation and activates HPV16 late polyadenylation over time. (**D**) RT-PCR with primers that specifically detect the two alternatively spliced HPV16 L1 mRNAs named L1 and L1i. RT-PCR primers are indicated in (**B**). The DNA damage response induced with 50 uM melphalan alters HPV16 mRNA splicing and results in efficient inclusion of the central exon on the HPV16 L1 mRNA.

The HPV E2 protein binds to the HPV DNA genome and together with HPV E1 it is required for replication of the HPV genome [14,15]. As the E2 protein accumulates to high levels in the HPV infected cells, E2 binds to multiple sites in the HPV early promoter to shut it down [14], thereby inhibiting E6 and E7 expression and allowing the cell to resume differentiation. Cell differentiation activates the late, differentiation-dependent HPV promoter [16], thereby paving the way for late L1 and L2 expression. The HPV E2 protein also has an inhibitory effect on the HPV early polyadenylation signal, possibly through interactions with CPSF30, and can cause read-through into the HPV late region of the genome [55]. Thus, E2 has a dual role in the HPV life cycle: it functions in HPV DNA replication and in the regulation of HPV gene expression. Recruitment of E2 to the DNA genome is required for HPV DNA replication and HPV E2 contributes to induction of HPV late gene expression by inhibiting the HPV early polyadenylation signal pAE. Similar to HPV E2, DDR factors are recruited to the HPV DNA genome and they are required for replication of the HPV genome [56]. It has recently been shown that activation of the cellular DDR also involves recruitment of RNA processing factors [57,58,59]. Thus, it was reasonable to speculate that DDR factors already recruited to the HPV genome also contribute to induction of HPV late gene expression, especially since HPV late gene expression occurs immediately following HPV genome replication. Furthermore, it has been recently shown that the cellular DDR interacts with RNA processing factors [57,58,59,60] and that the cellular DDR affects alternative splicing of cellular mRNAs [61,62,63,64]. To test the idea that the DDR contributes to HPV late gene expression, we used reporter cell line C33A2 that is designed to study induction of HPV16 late gene expression to investigate if the DNA damage response could activate HPV16 late gene expression [53,65,66]. Addition of the DNA damaging agent melphalan to this reporter cell line efficiently induced the DNA damage response in the C33A2 cells, and efficiently activated the HPV16 late *L1* and *L2* gene expression [66]. We observed a several hundred-fold induction of HPV16 L1 and L2 mRNAs as a result of inhibition of HPV16 early polyadenylation and activation of HPV16 L1 mRNA splicing, while the effect at the level of transcription was relatively modest [66]. Figure 4 shows the striking shift from early polyA site usage in HPV16 to primarily late polyA signal usage in response to induction of the DDR (Figure 4). Thus, the DDR induced HPV16 late gene expression at the level of HPV16 RNA processing, primarily by altering HPV16 splicing and polyadenylation [66]. The DDR factors BRCA1, Chk1, Chk2 and ATM were phosphorylated in response to DNA damage, as expected. Inhibition of ATM- or Chk1/2-phosphorylation, but not ATR-phosphorylation, prevented induction of HPV16 late gene expression [66], demonstrating that activation of the DDR contributed to induction of HPV16 late gene expression at the level of RNA processing. 

### 2.6. Cellular DNA Damage Response Factors Associate with HPV16 DNA and Recruit Cellular RNA Processing Factors

Inducing the DNA damage response in the C33A2 reporter cell line for HPV16 late gene expression resulted in recruitment of DDR factors BRCA1, in particular phosphorylated BRCA1, and BARD1 to the HPV16 DNA [66]. In addition, the more elusive BCLAF1 [67] and TRAP150 [68,69] proteins were also recruited to the HPV16 DNA [66]. Although BCLAF1 is bound to general splicing factor U2AF65 in both the absence and presence of DNA damage, it is associated with phosphorylated BRCA1 only in response to DNA damage [66]. These results suggested that interactions between BCLAF1 and phosphorylated BRCA1 occurred in response to DNA damage and resulted in recruitment of splicing factor U2AF65 to the HPV16 DNA. In addition, phosphorylated BRCA1 interacted with general splicing factor SF3b in the presence of DNA damage. The close relative of BCLAF1 named TRAP150 associated with HPV16 DNA and interacted with splicing factor U2AF65 only in the presence of DNA damage, suggesting that also TRAP150 recruits U2AF65 to HPV16 DNA. However, in contrast to BCLAF1, TRAP150 appeared to recruit U2AF65 independently of phosphorylated BRCA1. Indeed, the splicing factor U2AF65 was increasingly associated with HPV16 DNA in response to DNA damage [66]. Other studies indicate that TRAP150 binds U2AF65 directly [66]. We also observed an increased association between the HPV16 DNA of phosphorylated SR-proteins in response to DNA damage [66]. Serine and arginine-rich (SR) proteins are well known for their splicing regulatory functions [70,71] and several SR proteins have been shown to control HPV mRNA splicing [18,19,20,22,51]. The effect on HPV16 alternative splicing is best shown by the increased inclusion of the exon located between SA3358 and SD3632 in the L1 mRNAs (Figure 4). Taken together, DDR factors that are associated with HPV16 DNA may recruit splicing factors to the HPV16 DNA, thereby increasing the chances that they associate with de novo synthesized HPV16 mRNAs and affect HPV16 mRNA processing.

In addition to recruiting general splicing factors and SR proteins to the HPV16 DNA, the DDR factors also recruited other cellular RNA binding proteins, e.g., heterogenuos ribonuclearprotein C (hnRNP C) [66]. This protein has previously been shown to induce HPV16 late gene expression and affect L1 mRNA splicing in just the same way as induction of the DDR did [72]. Phosphorylated BRCA1 interacted with hnRNP C only in response to DNA damage and hnRNP C increasingly associated with HPV16 DNA in response to DNA damage [66]. hnRNP C has been shown to co-localize with sites of DNA damage as part of the BRCA1-, BRCA2- and PALB2-complex in response to DNA damage [57]. This hnRNP C-containing complex affected mRNA splicing. Combined, these results suggested that phosphorylated BRCA1 recruited hnRNP C to the HPV16 DNA and that this recruitment increased the chances that hnRNP C would bind newly synthesized HPV16 mRNAs and potentially alter HPV16 alternative splicing.

### 2.7. Increased Association between HPV16 mRNA-Binding Proteins and Cellular Polyadenylation Factors in Response to DNA Damage

**Figure 5:**
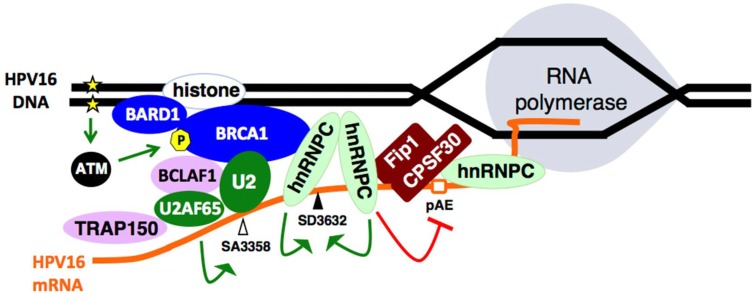
The DNA damage response activates HPV16 late gene expression by altering HPV16 mRNA splicing and polyadenylation. The DNA damage response induces ATM signalling. Activated ATM phosphorylates BRCA1, which leads to the formation of a pBRCA1-BCLAF1 complex that is recruited to the HPV DNA. The pBRCA1-BCLAF1 complex associates with splicing factors SF3b and U2AF65 and recruits them to the HPV16 DNA, thereby positioning the spliceosome in a strategic position for efficient detection of nascent HPV16 mRNA. hnRNP C is also recruited to both HPV16 DNA and mRNA through interactions with phosphorylated BRCA1. hnRNP C binds to the HPV16 early untranslated region and inhibits the HPV16 early polyadenylation signal pAE, possibly through interactions with Fip1 and CPSF30. This inhibition causes read-through at the early polyadenylation signal (pAE) and activates HPV16 late gene expression. hnRNP C also regulates HPV16 alternative splicing by activating late L1 splice site SD3632, contributing to the production of splices late L1 mRNAs. When the DNA damage response is activated, levels of the splicing regulatory protein TRAP150 increased in affected cells. The DNA damage response promotes the association of TRAP150 HPV16 DNA as well as with general splicing factor U2A65, thereby recruiting U2AF65 to HPV16 and contributing to the enhanced association of U2AF65 with HPV16 mRNAs. Taken together, the DNA damage response-induced associations of DNA damage response factors with RNA processing factors and with the HPV16 DNA and mRNAs alters HPV16 splicing and polyadenylation to induce HPV16 late gene expression.

The association between hnRNP C and polyadenylation factors CPSF30 and Fip1 increased in response to DNA damage, as did the binding of hnRNP C to the HPV16 early untranslated region [66]. Both hnRNP C and Fip1 binds to the U-rich region in the HPV16 early 3′-untranslated region (UTR) [72,73]. This suggested that hnRNP C contributed to inhibition of the HPV16 early polyadenylation signal pAE by binding to HPV16 mRNAs and negatively interfering with the polyadenylation factors Fip1 and CPSF30. Overexpression of hnRNP C with HPV16 subgenomic plasmids caused inhibition of the HPV16 early polyadenylation signal [66]. Knock-down or inhibition of CPSF30 inhibited the HPV16 early polyadenylation signal, but not the downstream late HPV16 polyadenylation signal [66]. In addition to hnRNP C, HuR binding to HPV16 early 3′-UTR increased in response to DNA damage [66]. HuR has been shown to inhibit HPV16 early polyadenylation and to contribute to export of HPV16 late mRNA from the nucleus [74]. Combined, these results support a model in which DDR factors assemble on HPV16 DNA and recruit RNA binding proteins including hnRNP C and HuR that bind to the HPV16 mRNAs. hnRNP C binds to polyadenylation factors CPSF30 and Fip1 to inhibit HPV16 early polyadenylation, thereby causing read-through into the late L1 and L2 coding region and activating HPV16 late gene expression (Figure 5).

### 2.8. DNA Damage Response Factors Recruit Splicing Factors to HPV16 DNA That Alter Splicing of HPV16 mRNAs

In addition to its role in HPV16 early polyadenylation, hnRNP C has also been shown to activate the suppressed HPV16 late splice site SD3632 to produce L1 mRNAs over the alternatively spliced L1i mRNA (see Figure 1 for structures of the HPV16 L1 and L1i mRNAs) [72]. This effect of hnRNP C is dependent on the HPV16 early UTR to which hnRNP C binds. Activation of HPV16 SD3632 results in L1 mRNAs in which the central exon between SA3358 and SD3632 is included on the mRNA as opposed to L1i mRNAs on which this exon is excluded. This effect of hnRNP C on HPV16 L1 mRNAs reproduced the effect of the DDR on alternative splicing of HPV16 L1 mRNAs (Figure 4) [66,72]. Thus, the hnRNP C proteins that were recruited to HPV16 DNA and to HPV16 mRNAs interacted with the HPV16 early UTR and inhibited HPV16 early polyadenylation and activated HPV16 L1 mRNA-specific late splice site SD3632. hnRNP C also suppresses polyadenylation of cellular mRNAs [75]. It is also of interest to note that hnRNP G, which is an RNA binding protein that plays an active role in the DDR [58], also controls HPV16 L1 mRNA splicing [76]. In conclusion, DDR factors recruit hnRNP C to the HPV16 DNA, thereby promoting association of hnRNP C with de novo synthesized HPV16 mRNAs. Consequently, splicing and polyadenylation of the HPV16 mRNAs are altered to favour HPV16 late gene expression.

Induction of the DNA damage response also resulted in enhanced splicing to HPV16 E2 splice site SA2709 and the HPV16 E4 splice site SA3358 [66]. While it is currently unknown how the E2 splice site is regulated, splice site SA3358 is controlled by splicing factors from the SR protein family including SRSF1, SRSF3 and SRSF9 [22,77,78,79,80,81]. The area at and around HPV16 splice sites SA3358 and SD3632 are hot-spots for cellular RNA binding proteins [82]. Enhanced splicing to SA3358 would explain the increase in the HPV16 E4 mRNAs spliced from SD880 to SA3358 as well as the enhanced production of the L2 mRNAs following activation of the DNA damage response [66]. It is reasonable to speculate that increased splicing to SA3358 is mediated by the enhanced association of phosphorylated SR proteins with the HPV16 mRNAs in response to DNA damage and/or the enhanced association of HPV16 mRNAs with general splicing factor U2AF65 [66]. In conclusion, activation of the DNA damage response results in the association of DDR factors with HPV16 DNA. These factors recruit various RNA binding proteins and RNA processing factor that alter HPV16 mRNA splicing and polyadenylation in a manner that favours HPV16 late gene expression. Thus, DNA damage response factors control HPV gene expression at the level of RNA processing in addition to their role in HPV DNA replication. Combined, the results suggest a model for activation of HPV16 late gene expression with the aid of the DDR that is presented in Figure 5.

### 2.9. The DNA Damage Response Affects Alternative Splicing of Cellular mRNAs

**Figure 6:**
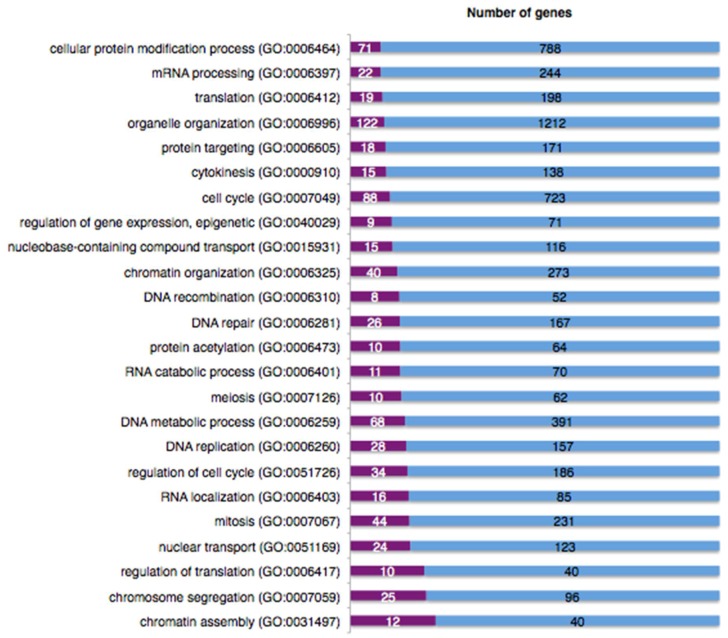
The DNA damage response affects mRNA levels of cellular genes with various biological functions, including genes encoding mRNA processing factors. Total RNA was harvested from HPV16 reporter cell line C33A2 after induction of the DNA damage response with 100 µM melphalan for 22 h. The RNA samples obtained from DMSO- or melphalan-treated C33A2 cells were subjected to microarray analysis to detect changes in mRNA levels throughout the genome. Total RNA was prepared using Qiagen RNeasy Mini Kit (Qiagen, Hilden, Germany) according to the manufacturer’s protocol. The RNA quality was determined using a Bioanalyzer (Agilent, Santa Clara, CA, USA). In total, five RNA samples each from melphalan and DMSO treated cells were analysed on Affymetrix GeneChip Human Transcriptome array 2.0 at SCIBLU Genomics (Lund University, Lund, Sweden). Protein coding genes that displayed at least a 2-fold change in mRNA levels between melphalan and DMSO treated cells, were sorted in the Transcriptome Analysis Console (TAC) from Thermo Fisher, Waltham, MA, USA). Following sorting, these genes were exported to PANTHER version 13.1, Gene List Analysis tool (Available online: http://pantherdb.org) for an overrepresentation test based on their biological function. Results of the RNA array analysis of RNA from DMSO or melphalan treated C33A2 cells are displayed as percentage of genes in each category that were either up- or down-regulated more than two-fold. The blue area shows the total number of genes in each biological-function category, and the purple area the number of genes that displayed a higher than two-fold change in mRNA levels between DMSO and melphalan treated cells.

**Figure 7:**
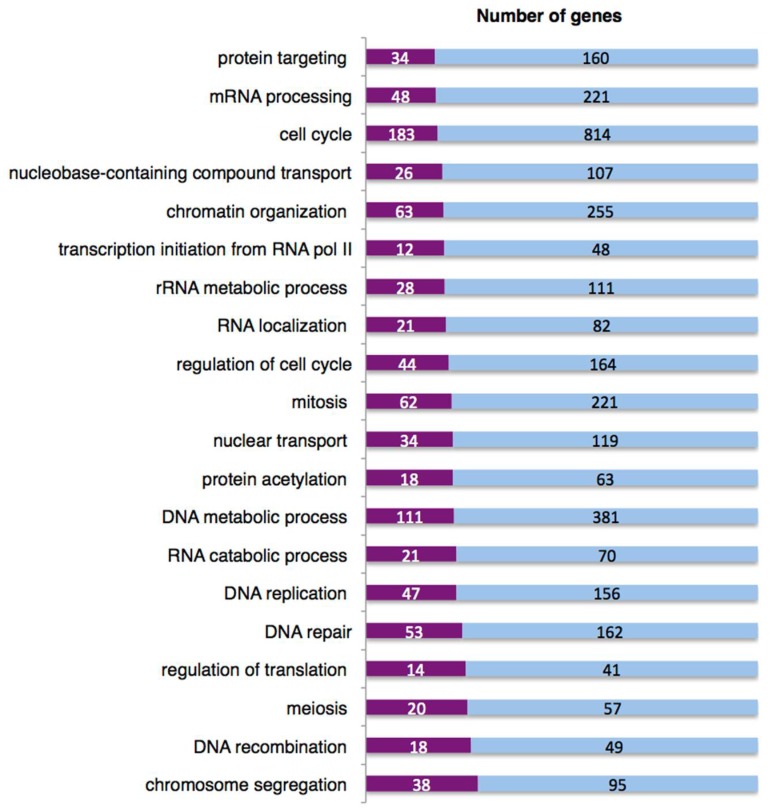
The DNA damage response induced by Melphalan affects splicing of cellular genes with various biological functions, including genes encoding mRNA processing factors. The data set obtained with the Affymetrix GeneChip Human Transcriptome array 2.0 and described in the legend of Figure 6 was analysed with the Transcriptome Analysis Console software (TAC) from Thermo Fisher. Protein coding genes with at least one two-fold change in the use of a splice junction or exon inclusion were exported into PANTHER, version 13.1, Gene List Analysis tool (Available online: http://pantherdb.org) for an overrepresentation test based on their biological function. Results of the RNA array analysis of DMSO or melphalan treated C33A2 cells are displayed as percentage of genes in each category that displayed altered splicing upon melphalan treatment. The blue area represents total number of genes in each biological category, and the purple area represents the number of genes producing mRNAs with altered alternative splicing in response to melphalan.

Given the ability of RNA binding proteins to interact with both chromatin and nascent mRNA, they could contribute to the response to DNA insult and to maintenance of the DDR signal. It has been shown that the BRCA1-BCLAF1 complex may position the spliceosome on genes for proper processing of transcripts in response to ATM/ATR signalling [83]. Indeed, we found that pBRCA1 and BCLAF1 were recruited to HPV16 chromatin and that they loaded splicing factors and RNA binding proteins onto HPV16 mRNAs [83]. Further, apart from activating HPV16 late gene expression at the level of RNA processing, induction of the DDR with melphalan also affected expression of many cellular genes as determined by an array analysis. Transcriptional changes in cellular genes not only affected DDR-genes, but also included genes coding for proteins involved in mRNA processing, RNA catabolic processes and RNA localization (Figure 6), suggesting that the DDR affected alternative splicing also of cellular mRNAs. As can be seen in Figure 7, up to 30% of the mRNAs in some gene groups showed changes in their alternative splicing in response to DDR activation (Figure 7).

Some of the mRNAs that were alternatively spliced in DDR-activated cells encoded DDR factors [66]. The HPV-infection alters the levels of many RNA binding proteins and splicing factors [84,85] and HPV16 E2 appears to indirectly affect splicing [23,86,87] as well as polyadenylation [55]. HPV infections may alter alternative splicing of cellular mRNAs through activation of the DDR. It has recently been shown that E6 and E7 increase transcription of HR-genes [88]. This effect could be due to the E6 and E7 effect on cellular transcription factors such as p53. p53 is one of the most well known examples of a mediator between the DDR response and RNA metabolism, effecting transcription and RNA turnover of many genes involved in the response to DNA damage. In addition, both E6 and E7 seem to induce cellular DSBs, independent of viral replication [88,89]. The amount of DSBs in viral and cellular DNA were the same until differentiation when active recruitment of HR-proteins to HPV DNA seemed to drive DSB repair, on the expense of cellular DSB repair [89]. As HPV has evolved to employ the DDR for genome amplification, it is possible that HPV gene expression has evolved in parallel to respond to the RNA processing factors brought to HPV DNA in complex with DDR factors.

## 3. Future Perspective

It is intriguing that DNA damage response factors can recruit RNA processing factors to the HPV16 DNA and that these RNA processing factors alter HPV16 mRNA splicing and polyadenylation in such a way that HPV16 late gene expression is activated [66]. These results warrant investigations of the connection between the DNA damage response and RNA processing in experimental systems that better illustrate the cell-differentiation-dependent HPV life cycle [90,91]. HPV16 gene expression is complex and involves regulation at the levels of transcription, splicing and polyadenylation [18]. Given that there are at least 10 different splice sites and two different polyadenylation sites that all compete with each other and are regulated by several different cellular RNA processing factors, it is conceivable that there are additional connections between the DDR and HPV mRNA processing.
